# Medical Affairs in Europe

**Published:** 1856-05

**Authors:** 


*Medical Affairs in Europe.*—On the Continent, during the past month, there does not seem much of novelty. M. Paul Dubois has been inquiring into an endemic of puerperal fever which has been prevalent in Paris, very inopportunely for the accouchement of the Empress Eugene, which occurs in a few days. From various communications with the French capital. and from meeting various Paris men recently in London, it seems that they now understand how to treat those cases better than they did. Dr. Ch. Martius has published a series of sketches recently (in the *Deutsche Klinik*), of what he has seen in London, and some of the views of Graves to "feed fevers," and of Dr. Todd to treat a whole host of inflammations in the same way, has caused an extraordinary sensation generally through France and Germany. Mr. Skey has been giving some most admirable lectures also in London, on chronic abscesses, in a surgical point of view; he believing they have their origin in a state of the system half fever, half inflammation, which he calls "below par," of which he has been giving a series of most brilliant exemplifications. Dr. Todd's erysipelatous poisoning of the blood from fever, Skey's "below par," and Graves's "feeding fever," have all a common origin; so much so, that Mr. Skey now tells his class they have learnt too much surgery, and they must give it up for medicine!

[*Dublin Med. Press.*

